# Two Cases of Diffuse Alveolar Haemorrhage Attributable to Human Metapneumovirus Infection: A Case Report

**DOI:** 10.1002/rcr2.70599

**Published:** 2026-04-28

**Authors:** Masamichi Lee, Junji Takiguchi, Yuu Kobayashi, Yoshihiko Tanimoto, Ai Mori, Hiromi Tomioka

**Affiliations:** ^1^ Kobe City Medical Center West Hospital Kobe Hyogo Japan; ^2^ Department of Infectious Diseases Kobe Institute of Health Kobe Hyogo Japan

**Keywords:** diffuse alveolar haemorrhage, human metapneumovirus, multiplex PCR, pneumonia

## Abstract

Diffuse alveolar haemorrhage (DAH) is a rare but potentially life‐threatening syndrome with diverse etiologies. Human metapneumovirus (hMPV) infection has rarely been reported as a cause of DAH. We report two cases of DAH associated with hMPV infection in immunocompetent adults. In both patients, bronchoalveolar lavage fluid (BALF) showed progressively haemorrhagic returns and hemosiderin‐laden macrophages, consistent with DAH. Multiplex polymerase chain reaction (PCR) using BALF detected hMPV, while extensive investigations excluded autoimmune, bacterial, and other viral causes. These cases suggest that lower respiratory tract infection with hMPV can be associated with DAH, even in immunocompetent adults. Screening for hMPV should be considered in cases of DAH of unknown aetiology, and multiplex PCR using BALF is a useful diagnostic tool.

## Graphical abstract

We report two cases of diffuse alveolar haemorrhage attributable to human metapneumovirus (hMPV), confirmed by PCR analysis of bronchoalveolar lavage fluid. Although the precise efficacy remains uncertain, both cases showed clinical improvement following short‐term corticosteroid therapy.

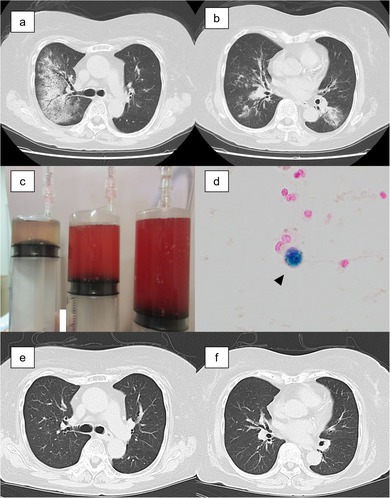

## 1 Introduction

Human metapneumovirus (hMPV), first identified in 2001, is an RNA virus that primarily infects children and immunocompromised patients and causes upper and lower respiratory symptoms [1]. The BioFire FilmArray Respiratory Panel (RP, bioMérieux, Marcy‐l'Étoile, France) rapidly detects multiple respiratory viruses, including hMPV and atypical bacterial targets, via nested polymerase chain reaction (PCR) [2]. On the other hand, while diffuse alveolar haemorrhage (DAH) is a potentially life‐threatening syndrome, hMPV‐associated DAH has only rarely been reported. This report describes two cases of adult DAH attributable to hMPV infection in which hMPV was detected in lower respiratory tract samples via RP.

## 2 Case Report

**FIGURE 1:**
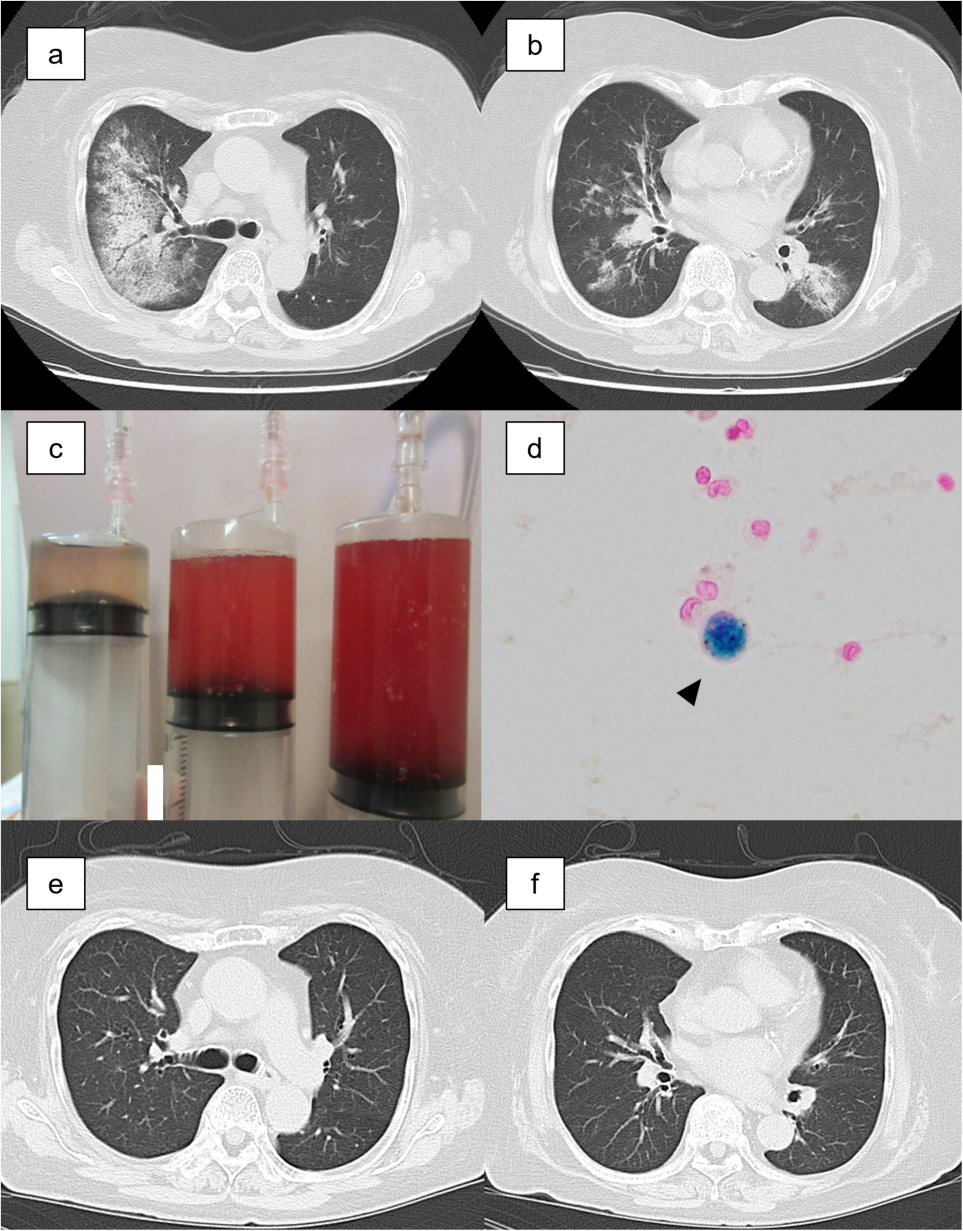
Test results of Patient A. Chest computed tomography (CT) on the day of admission. Infiltrative shadows were present in the right lung upper lobe (a) and lower left lobe (b). The patient's bronchoalveolar lavage fluid (BALF) appeared progressively bloodier. The three syringes, arranged from left to right, represent the first, second, and third collections of BALF, respectively (c). Berlin blue staining of BALF revealed hemosiderin‐laden macrophages (d; original magnification, ×400). CT at 1‐month post‐discharge. Bilateral upper and lower lobe infiltrates have resolved (e, f).

Patient A, a 79‐year‐old woman with a history of hypertension and dyslipidemia, presented with a 3‐day history of cough and visited a clinic. Chest computed tomography (CT) revealed infiltrative opacities in the right upper and left lower lobes (Figure 1a,b). Because community‐acquired pneumonia was suspected, she was given oral levofloxacin 500 mg once daily. However, because of persistent cough and haemoptysis, she was referred to our hospital 3 days later and admitted.

On admission, her vital signs were as follows: temperature, 36.8°C; blood pressure, 129/56 mmHg; pulse, 83 beats/min; and oxygen saturation, 94% with 3 L/min of oxygen via a nasal cannula. The patient's breath sounds decreased on auscultation of the right upper lung field. The laboratory findings included a normal white blood cell count and haemoglobin level, but her C‐reactive protein (CRP) level was elevated at 5.81 mg/dL (reference range: 0–0.5 mg/dL). Serological testing revealed positivity for antinuclear antibodies at titres of 1:80 (homogeneous pattern) and 1:40 (speckled pattern) but negativity for anti‐neutrophil cytoplasmic antibodies and anti‐glomerular basement membrane (GBM) antibodies. Urinalysis was unremarkable. RP version 2.1 revealed positivity for hMPV in nasopharyngeal swabs. Bronchoalveolar lavage (BAL) of the right upper lobe (B3) was performed via the instillation of 150 mL of saline and the retrieval of 64 mL of increasing amounts of bloody bronchoalveolar lavage fluid (BALF), which was consistent with DAH, as presented in Figure 1c. BALF cell analysis revealed 48.0% macrophages, 22.0% lymphocytes, and 30.0% neutrophils, and BALF cytology confirmed the presence of hemosiderin‐containing macrophages (Figure 1d). The bacterial culture of the BALF was negative. BALF was also positive for hMPV on RP.

**FIGURE 2:**
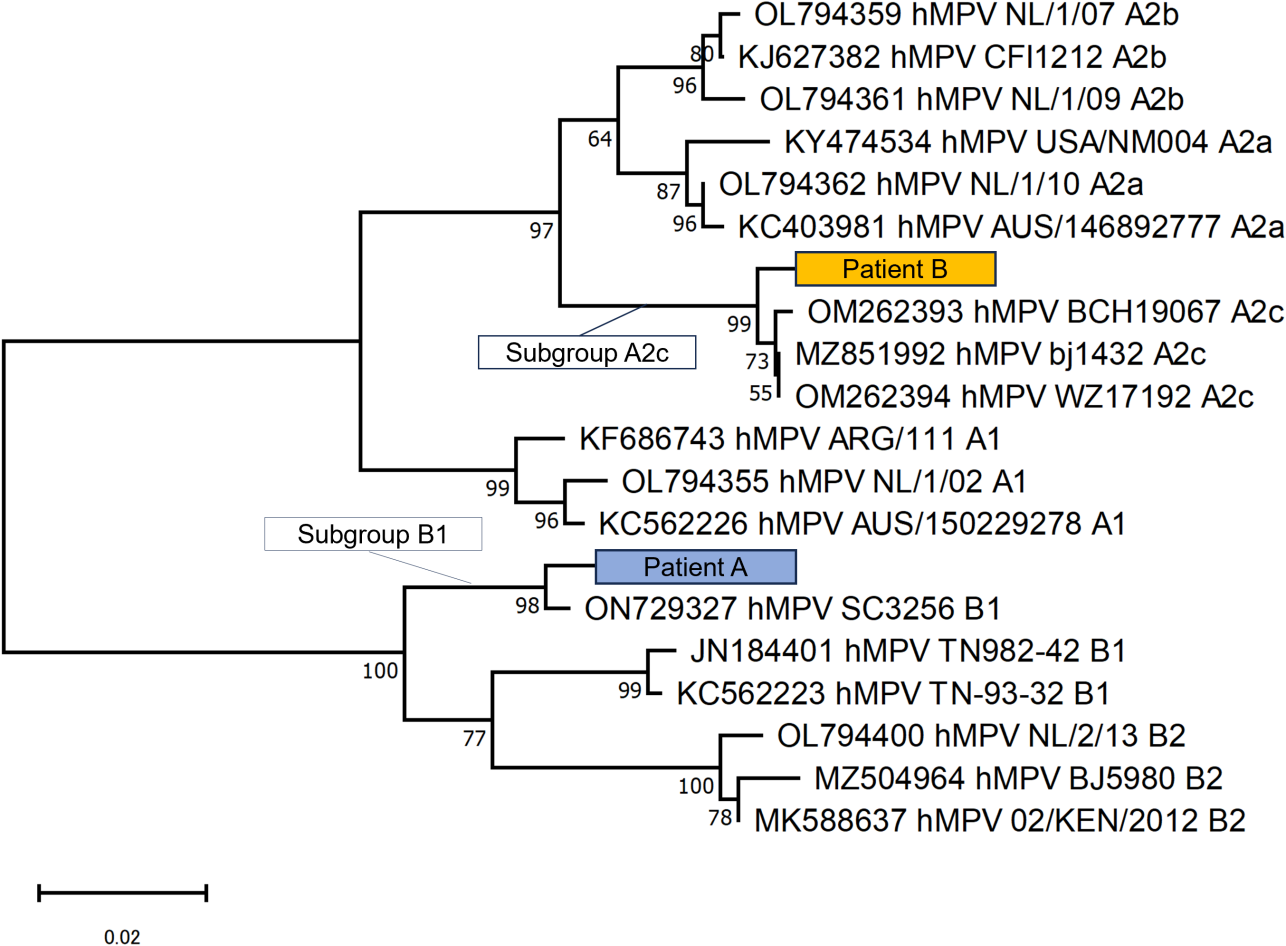
Phylogenetic analysis of the partial F gene (435 bp) of human metapneumovirus. Reverse transcription polymerase chain reaction and direct sequencing were performed via bronchoalveolar lavage fluid from patients with diffuse alveolar haemorrhage attributable to human metapneumovirus infection, including patients A and B. A phylogenetic tree was constructed via the neighbour–joining method with 1000 bootstrap replications. Bootstrap values (> 70) are indicated for the corresponding nodes. The scale bar indicates the number of nucleotide substitutions per site. Subtype B1 was detected in Patient A, whereas subtype A2c was detected in Patient B.

The patient was diagnosed with hMPV‐associated DAH, and she received intravenous methylprednisolone 500 mg daily for 3 days. Empiric antimicrobial therapy was initiated but discontinued early because of negative alveolar lavage cultures. On the fourth day of hospitalization, the patient's oxygen requirements began to decrease, and chest radiography demonstrated improvement in pulmonary infiltration. Her haemoptysis resolved on the sixth day of hospitalization. Supplemental oxygen was discontinued on the seventh day of hospitalization, and the corticosteroids were gradually tapered and stopped by the ninth day of hospitalization. The patient's clinical status improved, and she was discharged after 14 days of hospitalization. Subsequent reverse transcription (RT)‐PCR and phylogenetic analysis of the fusion (F) gene of hMPV in BALF revealed hMPV subtype B1 (Figure 2), although viral cultures were negative. Chest CT 1 month after discharge showed improved bilateral pulmonary opacities (Figure 1e,f).

**FIGURE 3:**
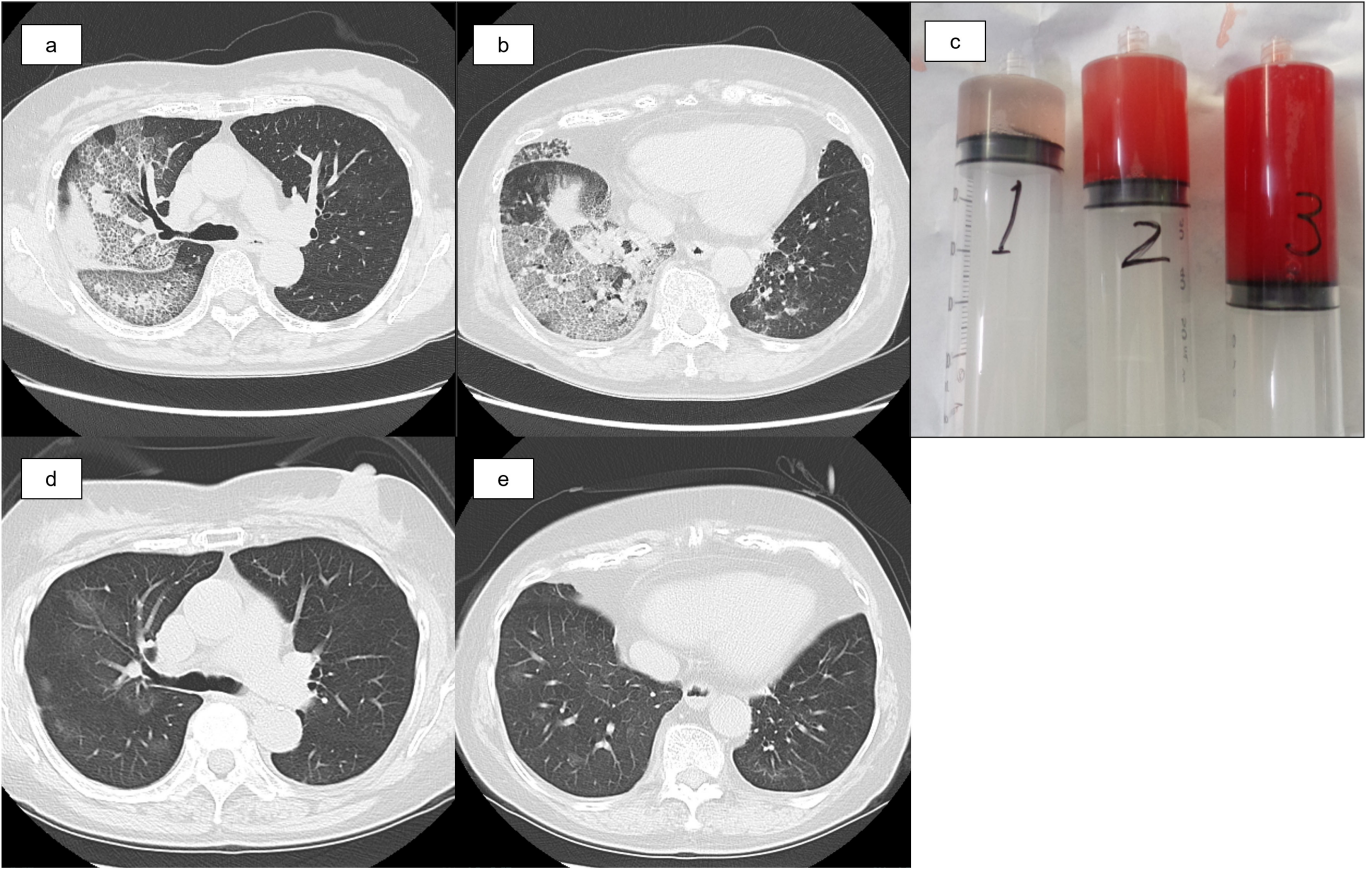
Test results of Patient B. Chest CT on the day of admission. Infiltrative shadows with ground‐glass opacities and interlobular septal thickening were observed in the right upper and lower lobes (a), and they were also present in the right lung middle lobe (b). Small areas of ground‐glass opacity were also observed in the lower lobe of the left lung (b). The patient's BALF appeared progressively bloodier. The three syringes, arranged from left to right, represent the first, second, and third BALF collections, respectively (c). CT at 1‐month post‐discharge. Bilateral upper and lower lobe infiltrates have almost resolved (d, e).

Patient B, a 67‐year‐old woman with a history of hypertension and alcoholic liver dysfunction, presented to the clinic with a 1‐day history of cough and haemoptysis. Chest CT revealed infiltrative opacities with ground‐glass attenuation and interlobular septal thickening throughout the right lung, as well as mild ground‐glass opacity in the left lower lobe (Figure 3a,b). Given her respiratory symptomatology, she was admitted on the same day for further management.

On admission, her vital signs were as follows: temperature, 36.5°C; blood pressure, 156/78 mmHg; pulse, 75 beats/min; and oxygen saturation, 94% on 2 L of oxygen via a nasal cannula. Coarse crackles were auscultated in the right posterior lung fields. The laboratory data included a normal white blood cell count, haemoglobin level of 15.9 g/dL (reference range: 11.1–15.1 g/dL), and CRP level of 2.85 mg/dL, as well as negativity for antinuclear antibodies, anti‐neutrophil cytoplasmic antibodies, and anti‐GBM antibodies.

The day following admission, the patient underwent BAL of the right middle lobe of the bronchus (B4), including the instillation of 150 mL of saline and retrieval of 75 mL of progressively bloodier BALF, raising suspicion of DAH (Figure 3c). BALF analysis revealed 17.0% macrophages, 19.0% lymphocytes, and 64.0% neutrophils. Using RP version 2.1, the BALF was positive for hMPV, whereas the bacterial culture remained negative.

Following BAL, the patient's respiratory status temporarily worsened, necessitating high‐flow nasal cannula oxygen therapy at a flow rate of 40 L/min and a fraction of inspired oxygen of 0.45. She was diagnosed with hMPV‐associated DAH and treated with 1 g of methylprednisolone daily for 3 days. By hospital Day 5, the patient's oxygen requirements had decreased, and chest radiography demonstrated improvement. Her clinical condition continued to improve steadily, and she was discharged on hospital Day 17. Subsequent RT–PCR and phylogenetic analysis of the F gene of hMPV in BALF revealed hMPV subtype A2c (Figure 2), but the viral culture results were negative. Chest CT 1 month after discharge showed improved bilateral pulmonary opacities (Figure 3d,e).

## 3 Discussion

hMPV causes two main types of acute lower respiratory tract disease: primary viral pneumonia and bacterial coinfection following viral illness [1]. In contrast, we encountered two cases of DAH associated with hMPV infection. DAH is a rare but potentially life‐threatening syndrome characterized by intra‐alveolar red blood cells and fibrin with the eventual accumulation of hemosiderin‐laden macrophages [3]. Although autoimmune diseases are common causes, certain viral infections, such as influenza and dengue, have also been implicated, but hMPV has rarely been associated with DAH.

It is known that DAH manifests three histopathologic patterns; pulmonary capillaritis, bland pulmonary haemorrhage, and diffuse alveolar damage (DAD) [3]. Pulmonary capillaritis is the most common, and is consisted from interstitial neutrophilic predominant infiltration, fibrinoid necrosis of the alveolar and capillary walls, and leukocytoclasis resulting in impairment of the pulmonary capillary basement membrane. It is often seen in connective tissue diseases such as systemic vasculitides, anti‐GBM disease, systemic lupus erythematosus (SLE), rheumatic diseases et al. Bland pulmonary haemorrhage is characterized by haemorrhage into the alveolar spaces without inflammation or destruction of the alveolar structures. It is often seen in coagulation disorders, mitral stenosis, idiopathic pulmonary haemosiderosis, and also in anti‐GBM diseases and SLE. DAD is the underlying pathological process in acute respiratory distress syndrome (ARDS). Infections, cytotoxic drug therapy, radiation therapy, and various other factors can cause DAD. In our cases, the pathological background of DAH remains unclear.

Our cases highlight this rare pulmonary manifestation of hMPV infection, including radiographic infiltrates, progressively hemorrhagic BALF with hemosiderin‐laden macrophages, and hMPV RNA in BALF, as detected by RT–PCR. Extensive evaluation dismissed alternative infectious, autoimmune, or idiopathic causes. In contrast to previous limited reports based solely on nasopharyngeal sampling [4], our cases directly implicate hMPV in the pathogenesis of lower respiratory tract diseases. Regardless of the specific hMPV genotype identified (A2c and B1 in our cases), current evidence suggests that there are no major strain‐dependent differences in clinical severity [5]. Prompt initiation of systemic corticosteroids resulted in clinical improvement, although the optimal treatment requires further study.

Our report was limited by its small sample size, precluding definitive conclusions about causality. An underlying predisposition or abnormal immune status might have contributed to these findings. Larger prospective studies are needed to better characterize this rare complication and explore potential risk factors. Nevertheless, our findings underscore the importance of considering hMPV in the differential diagnosis of unexplained DAH, for which multiplex molecular testing of lower respiratory tract specimens might improve diagnostic yield.

## Author Contributions

Masamichi Lee and Junji Takiguchi wrote the manuscript. Yuu Kobayashi and Hiromi Tomioka provided revisions and advice on the manuscript. Yoshihiko Tanimoto and Ai Mori performed RT–PCR and phylogenetic analysis.

## Funding

The authors have nothing to report.

## Consent

The authors declare that written informed consent was obtained for the publication of this manuscript and accompanying images and attest that the form used to obtain consent from the patient complies with the Journal requirements as outlined in the author guidelines.

## Conflicts of Interest

The authors declare no conflicts of interest.

## Data Availability

The data that support the findings of this study are available on request from the corresponding author. The data are not publicly available due to privacy or ethical restrictions.
